# A Monument vs. a Coffin

**Published:** 1857-05

**Authors:** 


*A Monument vs. A Coffin.*—During the prevalence of the yellow fever last summer on the South side of Long Island, two noble young physicians fell a sacrifice to their zeal in the service of their afflicted neighbors. The names of Dubois and Crane are enrolled with the names of those moral heroes who have died battling the woes and sufferings of their fellow men. Their grateful townsmen of New Utretcht intend erecting a monument at a cost of twelve hundred dollars, in the graveyard where they lie side by side.

Among those who fled upon the outbreak of the terrible scourge in another district, was a physician, who had enjoyed the support of a good practice, abandoning his patients, two of whom were lying ill of the fever. He viewed from the distance of several hundred miles the ravages of death, and heard the cry of help from his deserted home. The fever subsided, and the timid ventured to return, among these our chicken hearted doctor. So one bright frosty morning this fall, the rusty hinges of his office windows creaked as they admitted light to his neglected apartment. The cobwebs were swept from the medicine bottles, which had stood for months untouched. "*Hydragyri, submurias et Pulvis Doveri,*" looking out from their dusty cases, would speak a rebuke that they had so long been left without being sent upon an errand of mercy; but all unconscious, the doctor rubbed his hands with the satisfaction that he would once more be employed.

Thus a day was spent in delightful anticipations; night came—so did the morning; but what horror was depicted upon the doctor's countenance as he looked from his window; for lo! there, suspended in the air, hangs a coffin; its polished surface and glittering plate, inscribed with his name and departure, reflecting the beams of the morning sun. Upon taking it down he found a note from his surviving townsmen inviting him to be at home that night, as they intended to visit him, and place his remains in the mahogany coffin sent him. We need scarcely add that the doctor, considering his remains in his own possession better off than in the hands of his "surviving neighbors," made tracks, and has not been seen since in that locality.—*Newark Daily Adv.* and *Amer. Med. Gaz.*

